# Medical Management of Well-Differentiated Pancreatic Neuroendocrine Tumors: From Conventional Therapies to Emerging Strategies

**DOI:** 10.3390/jcm15051713

**Published:** 2026-02-24

**Authors:** Min Je Sung, Namyoung Park

**Affiliations:** 1 Digestive Disease Center, CHA Bundang Medical Center, School of Medicine, CHA University, Seongnam 13496, Republic of Korea; mj1744@hanmail.net; 2 Department of Internal Medicine, Seoul National University Bundang Hospital, Seongnam 13620, Republic of Korea

**Keywords:** pancreatic neuroendocrine tumor, systemic treatment, somatostatin analogue, peptide receptor radionuclide therapy, molecularly targeted agents, targeted alpha therapy, theranostics

## Abstract

Grade 1–2 pancreatic neuroendocrine tumors exhibit considerable biological and clinical diversity, which translates into a broad range of available therapeutic approaches. Given the absence of a universally accepted treatment sequence, treatment selection requires a practical framework based on tumor biology and clinical presentation. Clinical management should be individualized by integrating the histologic grade, disease extent, symptom burden, and somatostatin receptor (SSTR) expression. For patients with low-volume, SSTR-positive, and clinically indolent disease (Ki-67 < 10%), long-acting somatostatin analogues, including octreotide and lanreotide, are commonly used as initial therapies to control hormonal symptoms and delay tumor progression. In patients with radiologic progression requiring systemic disease control, targeted agents such as everolimus and sunitinib represent established subsequent options, particularly when disease stabilization is the primary therapeutic goal. Peptide receptor radionuclide therapy with ^177^Lu-DOTATATE has demonstrated meaningful antitumor activity and is generally considered in patients with SSTR-positive tumors with progressive disease (Ki-67 ≥ 10%) or increasing tumor burdens, especially when tumor reduction is desirable. Combination cytotoxic chemotherapy, most notably the capecitabine–temozolomide (CAPTEM) regimen, remains an important consideration for patients with higher tumor burdens or more aggressive tumor biology. This review summarizes current evidence and provides a practical overview of treatment selection and sequencing for the systemic management of Grade 1–2 pancreatic neuroendocrine tumors, while also highlighting emerging therapeutic strategies, including targeted alpha therapy and SSTR2 antagonist-based approaches.

## 1. Introduction

Pancreatic neuroendocrine tumors (PanNETs) comprise approximately 1% of pancreatic neoplasms, yet their relatively indolent biology and favorable prognosis contribute to a prevalence that is disproportionately high relative to their incidence [1,2]. Well-differentiated Grade 1–2 PanNETs, in particular, offer a broad range of therapeutic options and pose unique challenges in treatment management [3,4].

The clinical course and treatment response of PanNETs vary significantly based on several factors, including hormone secretion status, Ki-67 index, pattern and extent of metastases, and somatostatin receptor (SSTR) expression [5]. Because patients often experience prolonged survival, treatment selection should balance durable disease control with cumulative toxicity, while also prioritizing symptom control in functional syndromes. Over the past decade, the introduction of somatostatin analogues (SSAs), targeted agents, cytotoxic chemotherapy regimens, and peptide receptor radionuclide therapy (PRRT) has significantly expanded the therapeutic options available for patients with advanced PanNETs [3,4].

**Figure 1:**
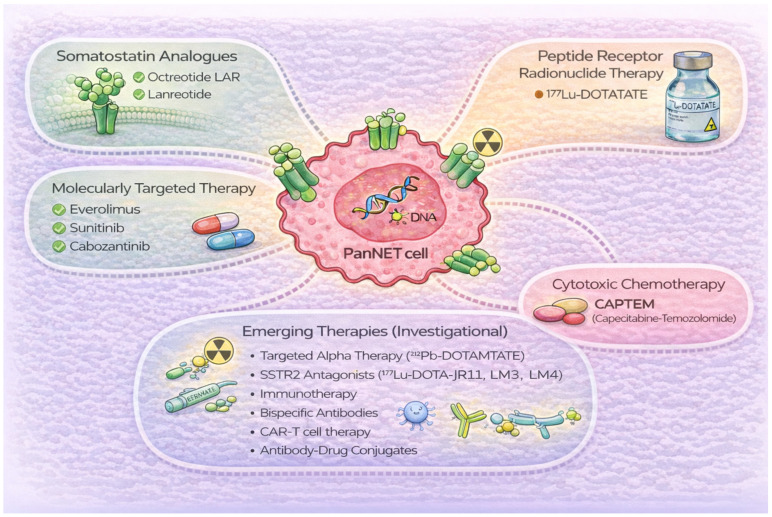
Summary of medical management of well-differentiated PanNETs. PanNET, pancreatic neuroendocrine tumor; LAR, long-acting release; CAPTEM, Capecitabine-Temozolomide; SSTR, somatostatin receptor; CAR-T, Chimeric Antigen Receptor T-Cell.

This review summarizes the current evidence and clinical utility of established systemic therapies for Grade 1–2 PanNETs, while also discussing innovative strategies such as targeted alpha therapy (TAT), SSTR2 antagonist-based PRRT, and other emerging approaches (Figure 1).

## 2. Treatment

### 2.1. Somatostatin Analogues (SSAs)

Somatostatin is a 14-amino-acid peptide that physiologically inhibits the secretion of multiple hormones. SSAs, such as octreotide and lanreotide, mimic these effects by binding to SSTRs expressed on the surface of most neuroendocrine tumors, thereby exerting antitumor and antisecretory activity [6]. The predominant pharmacologic effects of SSAs in PanNETs are mediated through SSTR2 and SSTR5. The antiproliferative mechanisms of SSAs involve both direct and indirect actions. Direct effects include inhibition of tumor cell proliferation through cell-cycle arrest and induction of apoptosis. Indirect effects include inhibition of tumor angiogenesis and suppression of hormones and growth factors that promote tumor growth, thereby modulating the tumor microenvironment and systemic milieu [7].

The presence and extent of SSTR expression can be assessed by molecular imaging using radiolabeled SSAs. Positron emission tomography/computed tomography with SSTR-targeted tracers, such as ^68^Ga-DOTATATE, is currently the preferred modality because of its high sensitivity and lesion detectability. In general, tumors demonstrating intense radiotracer uptake on SSTR-PET are more likely to show a favorable biochemical and antiproliferative response (approximately 60%) to SSA therapy [8]. However, in certain patterns of disease, such as diffuse miliary liver involvement or very small peritoneal deposits, functional SSTR expression may not be fully captured on imaging, and false-negative or under-estimated scans can occur despite biologically relevant receptor expression [9,10].

Functional PanNETs account for approximately 10–30% of all PanNETs [11]. In patients with SSTR-positive functional PanNETs, SSAs represent a key component of symptomatic management and quality-of-life preservation. The efficacy of symptom control is dose-dependent and varies by tumor subtype. Excellent responses have been reported in vasoactive intestinal peptide-secreting tumors (VIPomas) and glucagonomas. In contrast, the efficacy of SSAs in controlling insulinoma-related symptoms is less predictable. This is because SSAs can suppress counter-regulatory hormones, such as growth hormone, glucagon, and catecholamines, which may paradoxically exacerbate hypoglycemia. The data supporting the efficacy of SSAs in gastrinomas is limited [12,13,14,15].

Beyond symptom relief, SSAs are also capable of inhibiting tumor growth [16]. However, their antitumor benefit is mainly driven by disease stabilization and prolongation of progression-free survival (PFS), while objective radiographic tumor shrinkage is uncommon, historically reported in fewer than 10% of patients with solid gastroenteropancreatic neuroendocrine tumors (GEP-NETs) [17,18,19,20,21]. Nevertheless, multiple studies have consistently shown that SSA therapy delays disease progression and extends PFS, often alongside improvements in clinical symptoms [8,22,23]. Some analyses have further suggested that longer PFS achieved with SSA monotherapy is associated with overall survival (OS) [24]. Prognostic nomogram incorporating factors such as primary site, Ki-67 index, hepatic metastatic burden, presence of bone or peritoneal metastases, and documented progression status have been proposed to estimate PFS in patients with GEP-NETs [25].

The clinical rationale for octreotide LAR is largely supported by the PROMID trial, a placebo-controlled phase III trial (*n* = 85) in metastatic midgut NETs (Ki-67 ≤ 2%) with time to tumor progression (TTP) as the primary endpoint: median TTP was 14.3 months with octreotide LAR versus 6.0 months with placebo (*p* = 0.000072), and stable disease at 6 months occurred in 66.7% versus 37.2%, respectively [22]. In long-term follow-up, median OS did not differ significantly (84.7 vs. 83.7 months; hazard ratio (HR) 0.83; 95% confidence interval (CI) 0.47–1.46; *p* = 0.51), although extensive crossover to octreotide LAR in the placebo arm (38/43) likely confounded OS interpretation [26]. Importantly, it should be noted that the PROMID trial specifically studied midgut NETs and did not include PanNETs, limiting its direct applicability to PanNETs.

Lanreotide efficacy is supported by CLARINET, a randomized, placebo-controlled phase III trial (*n* = 204) in locally advanced or metastatic nonfunctioning pancreatic or intestinal NETs (Ki-67 ≤ 10%) with PFS as the primary endpoint: lanreotide significantly prolonged PFS compared with placebo (median not reached vs. 18 months; HR 0.47; 95% CI 0.30–0.73; *p* < 0.001) [8]. In the open-label extension interim analysis, estimated PFS with lanreotide was 32.8 months (95% CI 30.9–68.0) [27]. Differences in median PFS between PROMID and CLARINET studies likely reflect distinct trial populations, including a high proportion of patients with stable disease prior to randomization in CLARINET [8]. The clinical efficacy of systemic agents used in Grade 1–2 PanNETs, including SSAs, is summarized in Table 1.

For patients who develop radiologic progression after initial treatment with standard-dose SSAs, dose escalation of the same agent has been investigated as a potential strategy. The CLARINET FORTE study evaluated a high-frequency lanreotide regimen in patients with Grade 1–2 PanNETs and midgut NETs who had progressed on standard-dose lanreotide (120 mg every 4 weeks). In this trial, shortening the dosing interval to every 2 weeks yielded a median PFS of 5.6 months (95% CI 5.5–8.3) in the PanNET cohort [37]. Data from the control arms of the NETTER-1 (midgut NET) and NETTER-2 (GEP-NET) trials provide further clinical context regarding this dose escalation approach. In these prospective studies, patients assigned to the escalated dose octreotide LAR (60 mg) group demonstrated a median PFS of 8.4 months in NETTER-1 and 8.5 months in NETTER-2 [34,38]. However, the overall evidence supporting SSA dose escalation remains limited, and comparative data with other second-line options, such as PRRT or targeted agents, are lacking. Consequently, this strategy should be considered cautiously and individualized, taking into account the disease stage, alternative treatment options and therapeutic goals.

The choice between SSA formulations is largely determined by clinical convenience and patient preference. Octreotide LAR is administered via intramuscular injection, whereas lanreotide is given via subcutaneous injection once a month. Both agents demonstrate similar therapeutic efficacy, and selection is guided by factors such as cost, availability, preferred route of administration and tolerability. Comparative studies of patient preference have reported minimal injection-related pain with both formulations; some patients favored the subcutaneous administration of lanreotide, but no consistent or clinically meaningful differences were observed [39,40].

Overall, SSAs are well tolerated, and adverse events are generally mild to moderate [41,42]. During the first weeks of therapy, approximately one third of patients experience gastrointestinal adverse events such as nausea, abdominal discomfort, bloating, loose stools, or steatorrhea, which typically improve over time [8,22,41]. Additionally, injection-site pain and headache are also common adverse events [8]. Symptoms related to pancreatic exocrine insufficiency can be alleviated with pancreatic enzyme replacement when necessary. Furthermore, up to 25% of patients may develop asymptomatic cholelithiasis within 18 months of treatment initiation, likely as a consequence of reduced gallbladder contractility [8,22,42]. Notably, SSAs can induce transient glucose intolerance by inhibiting insulin secretion and reducing glucagon-like peptide 1 levels [43,44]. However, SSAs also suppress counter-regulatory hormones, including glucagon and growth hormone, thereby potentially reducing insulin resistance [45,46]. While hyperglycemia is a more common adverse event, hypoglycemia, though much less frequent, has been reported in up to 4% of patients in clinical trials [45].

### 2.2. Molecularly Targeted Therapy

Molecularly targeted therapy is a key option for patients with advanced or metastatic PanNETs, particularly after progression on SSAs or when tumor growth control is the primary therapeutic goal. The most widely used targeted agents currently include everolimus and multi-target tyrosine kinase inhibitors (TKIs), such as sunitinib and cabozantinib [28,29,30]. Belzutifan may also be considered in selected von Hippel-Lindau (VHL)-associated contexts [31,32].

#### 2.2.1. Everolimus

Everolimus is an oral mTOR inhibitor with antiproliferative and antiangiogenic effects. In RADIANT-3 (phase III; *n* = 410) in advanced progressive PanNETs, everolimus improved median PFS versus placebo (11.0 vs. 4.6 months; HR 0.35; 95% CI 0.27–0.45; *p* < 0.001) [28]. Partial responses were observed in 5% of patients receiving everolimus and 2% of those receiving placebo, with most of the clinical benefit arising from disease stabilization. Median OS was 44.0 months in the everolimus group and 37.7 months in the placebo group (HR 0.94; *p* = 0.30) [47]. The absence of a statistically significant difference in OS is generally attributed to the high rate of crossover, as approximately 85% of patients in the placebo group later received everolimus. Subgroup analyses showed that the PFS benefit of everolimus was consistent regardless of prior chemotherapy or concurrent use of SSA. Furthermore, clinically meaningful antitumor activity was observed even when everolimus was combined with SSAs [48]. Most drug-related adverse events were grade 1–2. The most frequently reported toxicities included stomatitis (64% vs. 17% with placebo), rash (49% vs. 10%), diarrhea (34% vs. 10%), fatigue (31% vs. 14%) and infections, predominantly of the upper respiratory tract (23% vs. 6%). Major grade 3–4 adverse events consisted mainly of stomatitis (7%), anemia (6%) and hyperglycemia (5%). Other studies have suggested that prior peptide receptor radionuclide therapy or cytotoxic chemotherapy may increase the risk of hematologic and pulmonary toxicity in patients subsequently treated with everolimus [49].

#### 2.2.2. Sunitinib

Sunitinib is an oral, multi-target TKI that inhibits several signaling pathways and growth factor receptors, including vascular endothelial growth factor (VEGF) [29]. In an initial phase II study, sunitinib 50 mg was administered on a 4-weeks-on/2-weeks-off schedule to 109 patients with advanced neuroendocrine tumors [50]. Among the 61 patients with PanNETs, 11 (18%) achieved a partial response, and 68% maintained prolonged stable disease; the median TTP was 7.7 months.

A subsequent randomized, double-blind, placebo-controlled phase III trial enrolled 171 patients with well-differentiated, progressive PanNETs and compared continuous daily sunitinib 37.5 mg with a placebo [51]. The study was stopped early because of clear evidence of benefit. Median PFS was 12.6 months in the sunitinib arm versus 5.8 months in the placebo arm. Eight objective responses were observed in the sunitinib group, including two complete responses. In long-term follow-up, median OS was 38.6 months with sunitinib and 29.1 months with placebo; however, this difference did not reach statistical significance, likely because 69% of placebo-treated patients crossed over to receive sunitinib [51].

Common adverse events include diarrhea, nausea, asthenia, vomiting, fatigue, erythrodysesthesia, hypertension, neutropenia, anorexia, stomatitis, dysgeusia, epistaxis, headache, insomnia, rash, and thrombocytopenia [29,52,53]. Class side effects of VEGF inhibitors, including hypertension and left ventricular dysfunction, require clinical consideration and management. In a meta-analysis of prospective studies in renal cell carcinoma, all-grade hypertension was observed in 22% of patients, with 7% experiencing severe hypertension [54]. Another meta-analysis including 6936 patients with regular cardiac monitoring reported an overall heart failure incidence of 4.1% (95% CI 1.5–10.6), with grade 3–4 events in 1.5% (95% CI 0.8–3.0) [55]. Sunitinib also has a dose-dependent effect on QTc prolongation [56,57,58,59]. Accordingly, clinical guidelines emphasize a comprehensive baseline cardiovascular risk assessment, including a detailed medical history, physical examination, and screening for major risk factors such as hypertension, diabetes, dyslipidemia, obesity, and smoking [60,61]. Close monitoring of blood pressure is essential, particularly during the initial phase of treatment and following dose escalations. Baseline electrocardiography (ECG) with QTc assessment is recommended, with serial follow-up ECGs for patients at moderate-to-high risk for QTc prolongation. Additionally, baseline echocardiography is recommended for high- and very high-risk patients and may be considered in low- and moderate-risk patients, with subsequent follow-up based on the patient’s cardiovascular risk [61].

Although the data are limited, case reports suggest that sunitinib can control refractory hormone-related symptoms in some patients with VIPoma [62]. Conversely, there are reports in which hypoglycemia did not improve or even worsened following sunitinib treatment in insulinoma, and cases of new-onset hypoglycemia have been described in initially non-functional PanNETs [63,64,65]. These observations underscore the importance of careful metabolic monitoring during therapy, particularly in patients with insulin-secreting tumors.

#### 2.2.3. Cabozantinib

Cabozantinib is an oral, multi-target TKI that inhibits MET, AXL, VEGFR2 and several other kinases. It is considered an important therapeutic option in patients with advanced PanNETs who have experienced disease progression after previous systemic treatments.

In the phase III CABINET trial (*n* = 298 NETs; PanNET *n* = 95) after progression on at least one prior systemic therapy, cabozantinib improved median PFS versus placebo (13.8 vs. 4.4 months; HR 0.23; 95% CI 0.12–0.42; *p* < 0.001) and increased objective response rate (ORR) (19% vs. 0%) [30]. Median OS was 40.0 months in the cabozantinib group and 31.1 months in the placebo group (HR 0.95), a difference that did not reach statistical significance. It is important to note that the overall survival data is still immature, as the study was terminated early due to the magnitude of the PFS benefit.

Treatment-related grade 3–4 adverse events occur in approximately 65% of patients receiving cabozantinib. The most common high-grade toxicities are diarrhea, hand-foot syndrome, fatigue and hypertension [30]. In a meta-analysis of VEGF TKIs, cabozantinib was associated with a nearly seven-fold increased risk of all-grade hypertension and showed the highest relative risk of high-grade hypertensive events among the agents evaluated [66]. Hypertension related to VEGF pathway inhibition is generally reversible and usually improves with treatment discontinuation [61,67]. Heart failure has also been reported, including severe and fatal cases [68,69]. Therefore, as with sunitinib, baseline cardiovascular risk assessment and active blood pressure monitoring are recommended during cabozantinib therapy, with ECG and echocardiographic evaluation guided by baseline risk [60,61].

#### 2.2.4. Belzutifan

Belzutifan is an oral, selective inhibitor of hypoxia-inducible factor 2α (HIF-2α), approved for VHL disease-associated tumors by United States Food and Drug Administration (US FDA), including renal cell carcinoma, central nervous system hemangioblastomas and PanNETs not requiring immediate surgery [70].

However, initial pivotal trials focused primarily on renal cell carcinoma, and patients with advanced or metastatic PanNETs were not included. In early analyses, among 61 patients with VHL disease, 22 had associated PanNETs, and an ORR of 91% was reported in these pancreatic lesions [32]. These responses, however, predominantly occurred in localized or low-volume diseases, and the efficacy of belzutifan in metastatic or rapidly progressive PanNETs remains uncertain. Furthermore, the role of belzutifan in patients with somatic VHL mutations, as opposed to those with germline VHL alterations, has not yet been clearly established [71]. It is important to note that belzutifan is not currently a standard treatment option for sporadic PanNETs, and its use remains limited to patients with VHL-associated PanNETs. The most commonly reported adverse events include anemia, fatigue, headache, dizziness, nausea, dyspnea, arthralgia, and constipation. Related clinical trials (NCT04924075) are currently ongoing.

### 2.3. Cytotoxic Chemotherapy

Cytotoxic chemotherapy retains an important role in the management of well-differentiated Grade 1–2 PanNETs, with comparatively high ORRs reported in several series (Table 1). In clinical practice, cytotoxic regimens are often prioritized in patients with a high tumor burden, rapidly progressive disease, or severe tumor-related symptoms, in whom prompt tumor shrinkage is desirable. Among the available regimens, the combination of capecitabine and temozolomide (CAPTEM) is the most widely used.

The evidence base for CAPTEM in PanNETs is largely derived from the ECOG-ACRIN E2211 randomized trial, which enrolled 144 patients with advanced, well-differentiated (Grade 1–2) PanNETs [36]. Patients were assigned to receive either temozolomide monotherapy or combination therapy with capecitabine and temozolomide. The CAPTEM arm demonstrated a significantly longer PFS compared with temozolomide alone (22.7 vs. 14.4 months; HR 0.58, 95% CI 0.36–0.93). ORR (40% vs. 34%) and disease control rate (DCR) (84% vs. 74%) were also higher with combination therapy. Median OS was longer in the CAPTEM group (58.7 vs. 53.8 months), although the difference did not reach statistical significance (HR 0.82, 95% CI 0.51–1.33). Treatment-related grade 3–4 adverse events were more frequent with CAPTEM than with temozolomide alone (44% vs. 22%), with neutropenia, nausea and vomiting, diarrhea and fatigue reported as the most common toxicities.

Beyond CAPTEM, several temozolomide-based combination regimens have been explored in smaller prospective studies, including combinations with thalidomide, bevacizumab and everolimus [72,73]. In addition, streptozocin- and oxaliplatin-based regimens have shown activity in PanNETs and are supported by a more limited body of evidence [74,75,76,77,78]. These alternative regimens may be considered in selected patients based on prior treatments, comorbidities, toxicity profiles and drug availability, but CAPTEM currently remains the most commonly used cytotoxic backbone in Grade 1–2 PanNETs.

### 2.4. Peptide Receptor Radionuclide Therapy (PRRT)

PRRT is an effective systemic treatment option for patients with SSTR-positive neuroendocrine tumors, including PanNETs. PRRT typically employs somatostatin analogues labeled with therapeutic radionuclides such as lutetium-177 (^177^Lu) or yttrium-90 (^90^Y). In contrast to conventional external-beam radiotherapy, which is mainly used for palliation in painful bone metastases, PRRT delivers high doses of ionizing radiation selectively to SSTR-expressing tumor cells in vivo, thereby achieving targeted internal radiotherapy.

Among available radioligands, ^177^Lu-DOTATATE is currently the most widely used. In 2018, the US FDA approved ^177^Lu-DOTATATE for the treatment of SSTR-positive GEP-NETs. The recommended regimen consists of 7.4 GBq (200 mCi) administered intravenously every 8 weeks for a total of 4 cycles.

The NETTER-1 trial (phase III; *n* = 229) in advanced SSTR-positive midgut NETs showed substantial benefit for ^177^Lu-DOTATATE plus standard-dose octreotide LAR (30 mg every 4 weeks) versus high-dose octreotide LAR (60 mg every 4 weeks) [38]. Although PanNETs were not included, NETTER-1 provided pivotal evidence of the antitumor efficacy and safety of ^177^Lu-DOTATATE in GEP-NETs as a class.

More directly relevant to PanNETs, the OCLURANDOM trial (phase II; *n* = 84) compared ^177^Lu-DOTATATE versus sunitinib in advanced SSTR-positive PanNETs without PRRT or TKI exposure [35]. At 12 months, PFS was 80.5% in the ^177^Lu-DOTATATE arm and 42% in the sunitinib arm. Median PFS was 20.7 months with ^177^Lu-DOTATATE and 11.0 months with sunitinib. Grade 3 or higher adverse events were observed in both groups (^177^Lu-DOTATATE, sunitinib), with fatigue (7% vs. 12%, respectively), decreased blood counts (12% vs. 24%), and hypertension (12% vs. 19%) being the most common toxicities.

The COMPETE trial (phase III; *n* = 309), presented at the Annual European Neuroendocrine Tumor Society (ENETS) meeting, compared ^177^Lu-edotreotide with everolimus in Grade 1–2 GEP-NETs [79]. Median PFS of 23.9 months in the PRRT arm, compared with 14.1 months in the everolimus arm (HR 0.67, 95% CI 0.48–0.95), while median OS did not differ significantly at the time of analysis (63.4 months vs. 58.7 months; HR 0.78, 95% CI 0.50–1.10; *p* = 0.206). Treatment-related adverse events were reported in 82.5% of patients receiving ^177^Lu-edotreotide and 97.0% of those receiving everolimus. Notably, one patient in the ^177^Lu-edotreotide arm developed myelodysplastic syndrome (MDS).

NETTER-2 trial (phase III; *n* = 226; 54% PanNETs) in high-risk SSTR-positive GEP-NETs (Ki-67 10–55%) demonstrated superior PFS for ^177^Lu-DOTATATE plus octreotide LAR 30 mg monthly versus high-dose octreotide LAR 60 mg monthly (median PFS 22.8 vs. 8.5 months; *p* < 0.0001), with consistent benefit in PanNET subgroups [34].

Real-world data also supports the efficacy of ^177^Lu-based PRRT in PanNETs. In a Dutch multicenter registry including 610 NET patients treated with cumulative activities exceeding 100 mCi, median PFS and OS were 29 months (95% CI 26–33) and 63 months (95% CI 55–72), respectively [80]. In the subset of 133 patients with PanNETs treated with ^177^Lu-DOTATATE, median PFS was 30 months, median OS was 71 months, and ORR was 54%. A meta-analysis comprising 887 patients treated with ^177^Lu-DOTATATE reported an overall ORR of 28% (95% CI 21–35%) and a DCR of 79% (95% CI 76–82%) in GEP-NETs [81].

^177^Lu-based PRRT is generally considered tolerable and safe. Common adverse events include fatigue, nausea, vomiting, hormonal disorders, nephrotoxicity, and cytopenia [33,34,35,80,81]. The most serious long-term toxicity associated with PRRT is irreversible myelotoxicity, including therapy-related myeloid neoplasms. These encompass MDS, acute leukemia, myeloproliferative neoplasms, and other secondary myeloid malignancies, and are associated with an unfavorable prognosis. Across clinical series, the incidence of MDS after PRRT has been reported at approximately 2–4%, whereas acute leukemia is consistently reported at <1% [80,82]. Risk appears to be influenced by advanced age, bone metastases, and extensive prior treatment exposure, although the contribution of prior alkylating chemotherapy remains debated [83,84]. Given the potential irreversibility of PRRT-associated hematologic toxicity, periodic complete blood count monitoring following PRRT is essential, with prompt hematology referral for persistent cytopenias or other hematologic abnormalities [82].

The optimal strategy for combining or sequencing PRRT with SSAs has not been fully defined. Current international guidelines, including those from the American Society of Clinical Oncology (ASCO) and the European Society for Medical Oncology (ESMO), recommend continuing SSA therapy after PRRT in patients with functional NETs, because carcinoid-related symptoms often do not completely resolve despite tumor control [3,4,17,82]. In contrast, for patients with non-functional NETs, the added value of SSA continuation beyond PRRT is less clear, and routine combination is not recommended in the absence of clear symptomatic or antiproliferative benefit [3,4].

## 3. Emerging Therapies

### 3.1. Targeted Alpha Therapy (TAT)

TAT offers a promising option compared with conventional beta-emitter PRRT, as alpha particles deposit high linear-energy transfer radiation, resulting in extremely localized and largely irreversible DNA double-strand damage [85]. Beta emitters, in contrast, mainly generate single-strand breaks, which tumor cells can more readily repair and may contribute to treatment resistance [86]. Because alpha particles have a short path length and limited penetration into surrounding tissue [87,88], they allow for highly selective irradiation of tumor deposits while sparing adjacent structures, an advantage that may be especially relevant in the treatment of hepatic metastases [89]. Despite encouraging early data, TAT is still in an investigational phase, and current clinical studies continue to evaluate its safety and therapeutic potential [90]. However, its clinical adoption is hindered by limited radionuclide availability, high production costs, complex logistics related to short isotope half-lives, and the need for highly precise targeting [91,92,93,94,95,96]. Therefore, streamlined production methods, and internationally standardized guidelines will be essential for incorporating TAT into routine clinical practice [96].

Ongoing trials are exploring the safety and efficacy of TAT in patients with SSTR-positive disease who have not previously received PRRT. The ongoing ALPHAMEDIX02 trial (phase II, NCT05153772) investigating ^212^Pb-DOTAMTATE demonstrated a high ORR of 57.1%, and high DCR of 85.7% [97]. Another ongoing Phase I/IIa trial (NCT05636618) assessing ^212^Pb VMT-alpha-NET suggests disease control in most patients completing planned cycles with median follow-up 17.4 months (range 9–26 months) [98,99].

### 3.2. Somatostatin Receptor 2 (SSTR2) Antagonists

In the field of SSTR-targeted therapy and imaging, the traditional understanding has been that the internalization of radiotracers is necessary to achieve effective binding and therapeutic outcomes. This concept has dominated the field for years, with agonists being considered the optimal choice for targeting SSTRs due to their ability to internalize into tumor cells. However, recent research has shown that this paradigm may not be entirely accurate. Ginj et al. demonstrated that SSTR2 antagonists may be more effective than agonists [100]. These antagonists bind more effectively to a larger number of receptor sites on the tumor cell surface without internalization, resulting in higher binding affinity and specificity [100,101]. This breakthrough has led to the development and investigation of several SSTR2 antagonists, which are currently investigational and being tested in both preclinical and clinical studies for their potential in targeted therapy and diagnostic imaging [102,103,104,105,106,107,108].

Among the most studied investigational SSTR2 antagonists, ^177^Lu-DOTA-JR11 stands out because of its ability to selectively deliver high radiation doses to SSTR2-positive tumors [103]. ^68^Ga-NODAGA-JR11, a companion imaging agent for ^177^Lu-DOTA-JR11, has demonstrated superior diagnostic performance compared with ^68^Ga-DOTATATE, with higher sensitivity (91.7% vs. 77.2%), detection of a greater number of lesions (1095 vs. 1003 lesions; *p* = 0.007), and a significantly improved target-to-background ratio in liver lesions (6.4 ± 8.7 vs. 3.1 ± 2.6, *p* = 0.000). ^68^Ga-NODAGA-JR11 also showed better image contrast, particularly in patients with low- to intermediate-grade GEP-NETs [104,105].

Other investigational SSTR2 antagonists, such as LM3 and LM4, have shown significant potential in both diagnostic imaging and therapeutic applications. In a first-in-human study, ^177^Lu-DOTA-LM3 demonstrated an impressive DCR of 85.1%, with partial responses observed in 36.2% of patients [102]. Treatment-related adverse events were limited to mild nausea (9.8%) and thrombocytopenia (5.9%), and no cases of severe nephrotoxicity, hepatotoxicity, or clinically relevant hematologic toxicity were reported, indicating that the treatment was generally well tolerated. In terms of imaging, both ^68^Ga-NODAGA-LM3 and ^68^Ga-DOTA-LM3 have demonstrated better diagnostic performance than ^68^Ga-DOTATATE, with increased tumor uptake and improved tumor-to-liver ratios, which contributed to better lesion detection and higher diagnostic accuracy [108]. Furthermore, LM4, a modified version of LM3, exhibited enhanced tumor retention and reduced renal uptake in preclinical studies, which may improve patient outcomes by minimizing toxicity and optimizing therapeutic delivery [106,107].

SSTR2 antagonists remain investigational, but early-phase clinical experience with SSTR2 antagonists suggests that hematotoxicity and therapy-related myeloid neoplasms warrant particular attention. In a phase I/II study of ^177^Lu-satoreotide tetraxetan grade 3 or higher hematologic toxicities were reported, including lymphopenia (7.5%), thrombocytopenia (7.5%), neutropenia (7.5%), and anemia (2.5%) [109]. In another phase I study, grade 3–4 leukopenia occurred in 10% after the first cycle, whereas prolonged grade 4 thrombocytopenia developed in 57% after a second therapeutic administration, often accompanied by grade 3 anemia (29%) and grade 3–4 neutropenia (43%) [110]. Notably, these severe cytopenias were observed even in patients with an estimated red marrow absorbed dose in the range of 1.5 Gy, a level commonly regarded as low in dosimetry-based safety assumptions. Mechanistic work has suggested that hematopoietic stem and progenitor cells (HSPCs) can exhibit SSTR2-ligand uptake comparable to NET cells [111]. Furthermore, SSTR2 antagonists show several-fold higher uptake in HSPCs than agonists. Because HSPCs constitute only a minute fraction of total bone marrow cells, their specific contribution may be difficult to capture with conventional imaging-based dosimetry. This discrepancy can lead to unexpectedly severe cytopenias despite seemingly acceptable whole-marrow dose estimates. In addition, therapy-related myeloid neoplasms were reported in the phase I experience, including acute myeloid leukemia (2.5%) and MDS (2.5%) [109]. Collectively, these findings support careful dose development and vigilant hematologic monitoring in ongoing and future SSTR2 antagonist trials.

### 3.3. Immunotherapy

The role of immunotherapy in PanNETs remains unclear. In the KEYNOTE-158 study evaluating the efficacy of the anti–programmed death 1 (PD-1) antibody pembrolizumab in patients with GEP-NETs, an objective response was observed in only 3.7% of patients. Notably, programmed death-ligand 1 (PD-L1) expression was negative even in responders [112]. In another phase Ib clinical trial investigating the anti–PD-1 antibody toripalimab, antitumor activity was observed in patients with recurrent or metastatic NETs; however, higher objective response rates were reported among patients with positive PD-L1 expression, high tumor mutational burden, and/or high microsatellite instability [113].

Nevertheless, combination immunotherapy appears to offer improved efficacy compared with monotherapy. A phase II study evaluating combined immune checkpoint blockade with anti-cytotoxic T-lymphocyte-associated antigen 4 (CTLA-4) (ipilimumab) and anti-PD-1 (nivolumab) in patients with rare cancers, including advanced neuroendocrine tumors, reported an ORR of 43%, with a median PFS of 4.8 months and a median OS of 14.8 months [114]. Similarly, the phase II DART trial investigated dual CTLA-4 and PD-1 blockade with ipilimumab and nivolumab in patients with NETs, demonstrating an ORR of 26% and a 6-month progression-free survival rate of 32% in the high-grade NET cohort [115].

In addition, an open-label, phase II basket trial involving patients with rare cancers, including PanNETs, assessed the combination of the immune checkpoint inhibitor atezolizumab and the angiogenesis inhibitor bevacizumab. This regimen demonstrated moderate clinical activity in patients with advanced NETs, with an ORR of 20% (4 patients) among those with pancreatic neuroendocrine neoplasms and a favorable safety profile, achieving median PFS of 14.9 months [116].

### 3.4. Bispecific Antibodies

Bispecific antibodies are engineered antibodies designed to simultaneously bind two distinct antigens, enabling targeted immune cell engagement or dual pathway modulation to enhance antitumor activity [117]. Tidutamab (XmAb18087) is a representative example of a bispecific antibody. It is a bispecific antibody targeting SSTR2 and CD3, which induces SSTR2-dependent T-cell-mediated cytotoxicity, as demonstrated in a nonhuman primate model [118]. Tidutamab is currently being evaluated in patients with neuroendocrine tumors in a clinical trial (NCT03411915) [119]. In addition, bispecific antibodies targeting delta-like ligand 3 (DLL3) in combination with CD2, CD3, or CD47 are currently under investigation, including BI 764532 (NCT04429087) [120], HPN 328 (NCT04471727) [121], and PT217 (NCT05652686) [122].

### 3.5. Chimeric Antigen Receptor T-Cell (CAR-T) Therapy

CAR-T therapies have recently gained increasing attention. CARs are genetically modified autologous T cells that are redirected ex vivo to recognize specific antigens expressed on the surface of cancer cells, thereby inducing cytotoxic activity and tumor lysis [123]. Approaches targeting cadherin-17 (CDH17), targeting SSTRs, and implementing adapter-based systems such as Octo-Fluo/AdFITC represent promising strategies for advancing CAR-T therapy in PanNETs [124,125]. Although PanNETs are not explicitly included, the efficacy and safety of genetically modified autologous T lymphocytes expressing CDH17 are currently being evaluated in a phase I/II clinical trial (NCT06055439) [126].

### 3.6. Antibody Drug Conjugate (ADC)-Based Modalities

ADCs are precision oncology platforms that chemically link highly potent cytotoxic drugs to tumor cell-targeting antibodies for selective drug delivery [127]. Studies targeting SSTR2, delta-like 1 homolog (DLK1), or DLL3 are currently underway. Among these, SSTR2-targeted approaches are being investigated at the preclinical stage. ADCT-701 is a DLK1-targeted antibody–drug conjugate currently being evaluated in a phase I clinical trial (NCT06041516) that includes patients with neuroendocrine tumors and neuroendocrine carcinomas [126]. ZL-1310 (zocilurtatug pelitecan) is a next-generation DLL3-targeted ADC currently being evaluated in a phase Ib/II clinical trial (NCT06885281) designed for selected solid tumors, with expansion to gastroenteropancreatic neuroendocrine carcinomas, including pancreatic primaries [128,129].

## 4. Symptom-Based Approaches for Functional PanNETs

In functional PanNETs, effective cytoreductive strategies using medical, surgical, or interventional approaches are important for relieving hormone-related symptoms. In addition, several non-cytotoxic therapies can suppress hormonal hypersecretion or alleviate its secretory effects, depending on the underlying hormone syndrome. In insulinomas, stabilizing glucose levels is important and can be achieved with dietary measures and diazoxide, with everolimus considered in selected patients [130,131,132]. For gastrinomas, high-dose proton pump inhibitors are the preferred acid-suppressive therapy [133,134,135]. SSAs are important options for symptom control in functional PanNETs and are commonly considered first-line therapy in VIPomas and glucagonomas [136,137,138]. In contrast, in insulinoma, SSAs should be used cautiously and only in SSTR-positive disease, as suppression of counter-regulatory hormones may paradoxically exacerbate hypoglycemia in selected patients [15]. Across these functional syndromes, short-acting octreotide may be utilized as a rescue therapy for breakthrough symptoms, supporting symptomatic stability alongside long-acting formulations [139].

## 5. Conclusions

Well-differentiated Grade 1–2 PanNETs exhibit highly heterogeneous clinical behavior. Accordingly, treatment decisions should be guided not only by the tumor grade and Ki-67 index but also by the functional status, tumor stage, metastatic burden, SSTR expression, and overall disease kinetics. In advanced or metastatic Grade 1–2 PanNETs, a broad spectrum of systemic options is now available, including SSAs, molecularly targeted agents, cytotoxic chemotherapy and PRRT. Although multiple guidelines have been published, a definitive, universally accepted treatment sequence has not been established. When these recommendations are synthesized, long-acting somatostatin analogues may be considered an initial therapeutic option for patients with unresectable, SSTR-positive Grade 1–2 pancreatic neuroendocrine tumors, particularly in those with low tumor burdens and clinically indolent disease. In patients with radiologic progression requiring systemic disease control after SSA therapy, targeted agents such as everolimus or sunitinib represent appropriate subsequent treatment options, especially when disease stabilization is the primary therapeutic goal or when tumors lack sufficient SSTR expression. Peptide receptor radionuclide therapy may be considered in patients with SSTR-positive tumors demonstrating progressive disease or increasing tumor burdens, particularly when tumor reduction is clinically desirable. For patients requiring rapid tumor shrinkage—such as those with a high tumor burden, rapidly progressive disease, or severe tumor-related symptoms—cytotoxic chemotherapy may be preferred, given its relatively high ORR.

However, the absence of robust predictive biomarkers limits the ability to tailor drug selection and sequencing to individual patients. To overcome these challenges, increasing attention is being paid to the discovery of novel molecular targets and the development of innovative therapeutic strategies, including TAT and SSTR2 antagonist-based approaches. Emerging therapeutic approaches remain largely in the preclinical or early-phase clinical stages of development and, therefore, require further clinical validation before they can be integrated into routine clinical practice [3,140].

Given this complexity, optimal management requires individualized treatment strategies, especially including SSTRs, tumor burdens, and symptoms. Beyond conventional PRRT, further therapeutic advances are anticipated with the development of TAT and SSTR antagonist-based PRRT. In parallel, the evolution of combination strategies, including PRRT in conjunction with SSAs, is expected to further enhance therapeutic efficacy. Moreover, ongoing advances in biomarker development may enable more precise treatment selection and support a more individualized therapeutic approach for patients with pancreatic neuroendocrine tumors.

## Figures and Tables

**Table 1:** Systemic therapy for grade 1–2 pancreatic neuroendocrine tumors.

Regimen	Treatment Arms	N	Primary Site	Grade	PFS Results	OS Results	Common Adverse Events
Octreotide LAR [22]	Arm A: Octreotide LAR 30 Arm B: Placebo	A: 42 B: 43	Midgut	G1	Median TTP A vs. B: 14.3 vs. 6.0 months HR 0.34 (95% CI 0.20–0.59) Stable disease at 6 months A vs. B: 66.7% vs. 37.2%	Median OS: N.E. HR 0.81 (95% CI 0.30–2.18); *p* = 0.77	Diarrhea, flatulence, bile stones
Lanreotide LAR [8]	Arm A: Lanreotide LAR Arm B: Placebo	A: 101 B: 103	Pancreas Other GI	G1/2 (Ki67 < 10%) G1: 69% G2: 20%	Median PFS A vs. B: NR vs. 18.0 months HR 0.47 (95% CI 0.30–0.73) 24 months PFS A vs. B: 65.1% vs. 33.0%	Not reported	Diarrhea, hyperglycemia, cholelithiasis, flatulence, injection-site pain, nausea, vomiting, headache, lethargy, decreased pancreatic enzymes
Everolimus [28]	Arm A: Everolimus Arm B: Placebo	A: 207 B: 203	Pancreas	G1: 83% G2: 16%	Median PFS A vs. B: 11.0 vs. 4.6 months HR 0.35 (95% CI 0.27–0.45) 18-month PFS A vs. B: 34% vs. 9%	Median OS A vs. B: 44.0 vs. 37.7 months HR 1.05 (95% CI 0.71–1.55); *p* = 0.59	Stomatitis, rash, diarrhea, fatigue, infections, anemia, hyperglycemia, thrombocytopenia, hypophosphatemia, neutropenia
Sunitinib [29]	Arm A: Sunitinib Arm B: Placebo	A: 86 B: 85	Pancreas	G1/2 Arm A Ki-67 > 5%: 36% Arm B Ki-67 > 5%: 45%	Median PFSa A vs. B: 11.4 vs. 5.5 months HR 0.42 (95% CI 0.26–0.66) 6-month PFS A vs. B: 71.3% vs. 43.2%	Median OS A vs. B: NR vs. NR HR 0.41 (95% CI 0.19–0.89); *p* = 0.02	Diarrhea, nausea, asthenia, vomiting, fatigue, erythrodysesthesia, hypertension, neutropenia
Cabozantinib [30]	Arm A: Cabozantinib Arm B; Placebo	A; 64 B; 31	Pancreas	G1; 22% G2; 61% G3; 12%	Median PFS A vs. B: 13.8 vs. 4.4 months HR 0.23 (95% CI 0.12–0.42) DCR at data-cutoff A vs. B: 50% vs. 19%	Median OS A vs. B: 40.0 vs. 31.1 HR 0.95 (95% CI 0.45–2.00)	Hypertension, fatigue, diarrhea, nausea, thromboembolic events
Belzutifan [31,32]	Single arm	22	VHL-mutated PanNETs	Not reported	Median PFS; NR (95% CI NR to NR)	Not reported	Anemia, fatigue, headache, dizziness, nausea, dyspnea, arthralgia, constipation
^177^Lu-Dotatate [33,34,35]	Arm A: ^177^Lu-Dotatate Arm B: Sunitinib	A: 41 B: 43	Pancreas	G1: 19% G2/3: 81%	12-month PFS A vs. B: 81% vs. 42% Median PFSa A vs. B: 20.7 vs. 11 months	Not reported	Fatigue, decrease blood count, hypertension
	Arm A: ^177^Lu-Dotatate + Octreotide LAR Arm B: Octreotide LAR	A: 151 B: 75	Pancreas Other GI	G2 65% G3 35%	Median PFSa A vs. B: 22.8 vs. 8.5 months HR 0.276 (95% CI 0.18–0.42) DCR at data cutoff A vs. B: 90.7% vs. 66.7%	Not reported	Nausea, diarrhea, abdominal pain, decrease blood count
CAPTEM [36]	Arm A: Temozolomide Arm B: Capecitabine-temozolomide	A: 72 B: 72	Pan	Arm A, G1/2: 38/62% Arm B. G1/2: 50/49%	Median PFSa B vs. A: 22.7 vs. 14.4 months HR 0.58 (95% CI 95% CI, 0.36 to 0.93); *p* = 0.023 DCR at end point A vs. B: 48% vs. 57%, *p* = 0.20	Median OS A vs. B: 53.8 vs. 58.7 months HR 0.8 (95% CI 0.51–1.33); *p* = 0.42	Fatigue, nausea, constipation, vomiting, headache, diarrhea, anorexia, abdominal pain, decrease blood count

CAPTEM, Capecitabine-Temozolomide; CI, confidence interval; GI, gastrointestinal; HR, hazard ratio; NR, not reached; LAR, long-acting release; OS; overall survival; PFS, progression-free survival; TTP, time to progression; PanNETs, pancreatic neuroendocrine tumors.

## Data Availability

No new datasets were generated or analyzed in this review.

## Abbreviations

PanNET	Pancreatic neuroendocrine tumor
SSTR	Somatostatin receptor
SSA	Somatostatin analogue
PRRT	Peptide receptor radionuclide therapy
TAT	Targeted alpha therapy
VIPoma	Vasoactive intestinal peptide-secreting tumor
PFS	Progression free survival
GEP-NET	Gastroenteropancreatic neuroendocrine tumor
OS	Overall survival
TTP	Time to tumor progression
HR	Hazard ratio
CI	Confidence interval
FDA	Food and Drug Administration
TTP	Time to progression
TKI	Tyrosine kinase inhibitor
VHL	von Hippel-Lindau
VEGF	Vascular endothelial growth factor
ECG	Electrocardiography
ORR	Objective response rate
HIF-2α	Hypoxia-inducible factor 2α
US FDA	United States Food and Drug Administration
CAPTEM	Capecitabine plus temozolomide
ENETS	European Neuroendocrine Tumor Society
MDS	Myelodysplastic syndrome
DCR	Disease control rate
ASCO	American Society of Clinical Oncology
ESMO	European Society for Medical Oncology
HSPC	Hematopoietic stem and progenitor cell
PD-1	Programmed death 1
PD-L1	Programmed death-ligand 1
CTLA-4	Cytotoxic T-lymphocyte–associated antigen 4
DLL3	Delta-like ligand 3
CAR-T	Chimeric antigen receptor T-cell
CDH17	Cadherin-17
ADC	Antibody-drug conjugate
DLK1	Delta-like 1 homolog
